# Molecular Epidemiology of HIV-1 in Ghana: Subtype Distribution, Drug Resistance and Coreceptor Usage

**DOI:** 10.3390/v15010128

**Published:** 2022-12-31

**Authors:** Anna Appah, Charlotte J. Beelen, Don Kirkby, Winnie Dong, Aniqa Shahid, Brian Foley, Miriam Mensah, Vincent Ganu, Peter Puplampu, Linda E. Amoah, Nicholas I. Nii-Trebi, Chanson J. Brumme, Zabrina L. Brumme

**Affiliations:** 1 Faculty of Health Sciences, Simon Fraser University, Burnaby, BC V5A 1S6, Canada; 2 British Columbia Centre for Excellence in HIV/AIDS, Vancouver, BC V6Z 1Y6, Canada; 3 Los Alamos National Laboratory, P.O. Box 1663, Los Alamos, NM 87545, USA; 4 Fevers Unit, Department of Medicine, Korle Bu Teaching Hospital, Accra P.O. Box KB 77, Ghana; 5 Department of Internal Medicine, Korle Bu Teaching Hospital, Accra P.O. Box KB 77, Ghana; 6 Noguchi Memorial Institute for Medical Research, University of Ghana, Accra P.O. Box LG 581, Ghana; 7 Department of Medical Laboratory Sciences, School of Biomedical and Allied Health Sciences, University of Ghana, Accra P.O. Box LG 25, Ghana; 8 Faculty of Medicine, University of British Columbia, Vancouver, BC V6T 1Z3, Canada

**Keywords:** HIV, HIV-1, subtype diversity, pretreatment drug resistance, coreceptor usage, molecular epidemiology, Ghana

## Abstract

The greatest HIV-1 genetic diversity is found in West/Central Africa due to the pandemic’s origins in this region, but this diversity remains understudied. We characterized HIV-1 subtype diversity (from both sub-genomic and full-genome viral sequences), drug resistance and coreceptor usage in 103 predominantly (90%) antiretroviral-naive individuals living with HIV-1 in Ghana. Full-genome HIV-1 subtyping confirmed the circulating recombinant form CRF02_AG as the dominant (53.9%) subtype in the region, with the complex recombinant 06_cpx (4%) present as well. Unique recombinants, most of which were mosaics containing CRF02_AG and/or 06_cpx, made up 37% of sequences, while “pure” subtypes were rare (<6%). Pretreatment resistance to at least one drug class was observed in 17% of the cohort, with NNRTI resistance being the most common (12%) and INSTI resistance being relatively rare (2%). CXCR4-using HIV-1 sequences were identified in 23% of participants. Overall, our findings advance our understanding of HIV-1 molecular epidemiology in Ghana. Extensive HIV-1 genetic diversity in the region appears to be fueling the ongoing creation of novel recombinants, the majority CRF02_AG-containing, in the region. The relatively high prevalence of pretreatment NNRTI resistance but low prevalence of INSTI resistance supports the use of INSTI-based first-line regimens in Ghana.

## 1. Introduction

HIV-1 remains a major global health concern, with Sub-Saharan Africa bearing 70% of the disease burden [1]. An estimated 84.2 million individuals worldwide have acquired HIV-1 since the beginning of the pandemic, with the cumulative death toll from HIV-related illness surpassing 40 million in 2021 [2]. Due to HIV’s extensive mutational and replicative capacity, its ability to establish lifelong infection, and the pandemic’s large scale, viral strains have substantially diversified over time, posing challenges to prevention and treatment [3,4,5,6,7]. To date, the HIV-1 group M (“pandemic”) strains are classified into ten subtypes (A–D, F–H and J–L) and 118 circulating recombinant forms (CRFs), designated when a particular recombinant has been detected in at least three epidemiologically unlinked persons [4,8,9]. The greatest HIV-1 genetic diversity is observed in West/Central Africa, primarily due to the virus’ origin in this region [10,11], but this diversity remains somewhat understudied. Note that, from now forward, we use “HIV” interchangeably with “HIV-1” to denote the HIV-1 group M pandemic strain.

Ghana reported its first HIV case in 1986 [12]. Since then, national HIV prevalence has remained consistent at ~2% [13,14]. An estimated 342,307 persons were living with HIV in Ghana in 2019 [15,16], the majority (65%) female [17,18], with heterosexual transmission representing the main transmission mode [19,20]. Though Ghana adopted the World Health Organization (WHO)’s “treat all” policy, which offers ART to all people living with HIV (PLWH) irrespective of their CD4+ T-cell count [21], in September 2016, the country fell short of achieving the UNAIDS “90–90–90” goals (where 90% of PLWH would know their status, 90% of those who know their status would be on antiretroviral therapy [ART], and 90% of those on ART would be virally suppressed, by 2020). As of 2018, an estimated 58% of Ghanaian PLWH knew their status, 78% of whom were on ART, and of whom 68% had suppressed viral load on ART [22]. 

ART use is also shifting in Ghana. Prior to July 2019, first-line regimens featured the nucleoside reverse transcriptase inhibitors (NRTIs) tenofovir  +  lamivudine (or emtricitabine) plus a non-nucleoside reverse transcriptase inhibitor (NNRTI), either efavirenz or nevirapine [23,24]. Second line regimens featured two NRTIs plus the boosted protease inhibitor (PI) atazanavir/ritonavir, while integrase strand transfer inhibitors (INSTI) were reserved for third line [23,24]. In July 2019 however, DTG-containing ART was introduced as the preferred first line regimen due to concerns over NNRTI resistance [25].

HIV molecular epidemiology studies in Ghana have been somewhat limited. The circulating recombinant form CRF02_AG dominates in the region, while “pure” subtypes (mainly A and G) as well as complex recombinants such as 06_cpx (a mosaic of subtypes A, G, J and K) and 09_cpx (comprising A- and G-like regions) also circulate at much lower frequencies [26,27,28]. The vast majority of Ghanaian HIV subtype data however are based on partial polymerase sequences that comprise only 10–15% of the viral genome [8,29], which may not represent the subtype of the full viral genome [30]. In fact, only 31 full-genome HIV sequences from Ghana currently exist in the public domain, all of which were obtained in 2003 or prior [8].

Drug resistance data are also somewhat limited in Ghana. Despite the WHO’s recommendation that routine drug resistance surveillance be conducted in settings where individualized drug resistance genotyping is not standard of care, the last HIV drug resistance survey in Ghana undertaken according to WHO guidelines occurred in 2013. Data from research studies however indicate that pretreatment resistance (defined as resistance in persons who discontinued ART more than three months ago without documented viral failure and who are now re-initiating first-line ART, or in treatment-naïve individuals), is increasing. While a study conducted on samples collected in 2003 from treatment-naïve Ghanaian PLWH reported no evidence of transmitted HIV drug resistance (TDR) [31], more recent studies have reported 9%, 11.5% and 33% TDR prevalence in children [32], pregnant women [33] and adults [27], respectively, though the number of individuals genotyped in these reports was relatively small. Acquired drug resistance is also a concern [28,32], with studies reporting 25–46% prevalence of the NNRTI resistance mutation K103N and a 39–54% prevalence of the NRTI resistance mutation M184V in persons failing ART [27,28,32,34]. Even fewer studies have investigated coreceptor usage, despite its relevance to the use of the HIV entry inhibitor maraviroc, which specifically inhibits viral entry via the CCR5 coreceptor (and is thus only effective in individuals who exclusively harbor CCR5-using HIV) [35]. A small phenotypic study of 27 symptomatic Ghanaian PLWH undertaken in 2007 indicated that CCR5 use predominated [36], but no other studies to our knowledge have investigated HIV co-receptor usage in the region. Data from other global regions also indicates that coreceptor usage distribution differs by HIV subtype [37,38,39], but this has not been investigated among the diverse subtypes circulating in Ghana.

To address these knowledge gaps, we characterized HIV subtype diversity, pretreatment drug resistance and coreceptor usage in a cohort of 103 PLWH in Ghana (90% of whom were ART-naive and 10% who had discontinued first-line ART at least two years prior), using a combination of Sanger and next-generation sequencing methods.

## 2. Materials and Methods

### 2.1. Study Design and Sampling

We recruited 103 PLWH (≥16 years) from major HIV care clinics in the Greater-Accra and Central regions of Ghana using purposive sampling in a cross-sectional design (2020–2022). To be eligible for inclusion, participants had to be either ART naïve or must have discontinued first line ART more than 2 years ago without evidence of treatment failure. Whole blood (6 ml) was collected by venipuncture from the forearm into ethylenediaminetetraacetic acid (EDTA) tubes. Blood was centrifuged the same day at 2000 G for 10 min to obtain plasma, which was stored at −20 °C until shipment on dry ice for HIV genotyping. Sociodemographic data, viral load and treatment records were collected by self-report and confirmed through medical records where available. 

### 2.2. Ethics Approval

This study was carried out in accordance with ethical regulations for research with human participants in line with the tenets of the Declaration of Helsinki. Each participant provided written informed consent. This study was jointly approved by the Simon Fraser University and Providence Health Care/University of British Columbia Research Ethics Boards in Canada (H19-01947), as well as the Institutional Review Board and the Scientific and Technical Committee of Korle-Bu Teaching Hospital, Accra, Ghana. (KBTH-IRB) 00075/2020.

### 2.3. HIV Genotyping: RNA Extraction and RT-PCR Amplification

Total RNA was extracted from 500 uL blood plasma using the NucliSENS^®^ EasyMag (bioMérieux, Montréal, QC, Canada) according to the manufacturer’s instructions, eluted in 60 ul, and stored at −80 °C until reverse transcription PCR (RT-PCR). A positive control (clinical sample) and aliquot of nuclease-free water were included in each extraction run as positive and negative controls, respectively, and carried through all subsequent RT-PCR reactions. The complete HIV coding region was bulk-amplified in five overlapping fragments, comprising gag-protease (GAGPR), protease-reverse transcriptase (PRRT), reverse transcriptase–viral protein u (RTVPU), viral protein r-glycoprotein120 (VPR-GP120) and glycoprotein41-negative factor protein (GP41Nef), using primers designed to capture circulating HIV diversity in Ghana, in particular subtypes A, G and CRF02_AG [29]. The primary and secondary (backup) primers used for RT-PCR are provided in Supplementary Tables S1 and S2. Note that the PCR primers did not feature unique molecular barcodes (primer IDs). Briefly, cDNA was generated using an HIV sequence-specific reverse primer and NxtScript Reverse Transcriptase by incubating at 42 °C for 45 min (Roche Diagnostics, Laval Canada). Nested PCR was then performed using the Expand HiFi system (Roche Diagnostics; Laval, Canada). Thermal cycling conditions for both rounds of PCR were; 94 °C for 2 min; 10 cycles of (94 °C for 15 s, 55 °C for 30 s, 72 °C for 2 min); 25 cycles of (94 °C for 15 s, 55 °C for 30 s and 72 °C for 2 min with an additional 5 s per cycle) and a final extension at 72 °C for 7 min. Amplicons were visualized on a 1% agarose gel. Samples failing PCR amplification were re-extracted at least twice, and amplification re-attempted using backup primers. 

#### 2.3.1. Sanger Sequencing of Pol Regions

Amplicons containing protease, reverse transcriptase and integrase regions were bi-directionally sequenced on an ABI Prism 3730xl DNA analyzer (Life Technologies, Burlington, ON, Canada) using the BigDye Terminator v3.1 cycle sequencing kit. Sanger sequencing primers are listed in Supplementary Table S3. Eight sequencing primers were used per amplicon to obtain at least twofold coverage. Chromatograms were called using RECall version 2.28.1, an in-house software that automatically calls bases, trims primer sequences, and constructs contiguous consensus sequences [40]. Nucleotide mixtures were automatically called if a subdominant peak of ≥17.5% of the total area of the dominant peak was observed in >50% of sequencing reads covering that position. 

#### 2.3.2. Whole HIV Genome Illumina Sequencing and Analysis

Samples for which all five overlapping HIV genome-wide RT-PCR reactions yielded amplicons were sequenced by Illumina MiSeq. Amplicon concentrations were normalized, and DNA was purified using AMPure XP magnetic beads (A63880, Beckman Coulter, Mississauga, ON, Canada) to ensure broadly equivalent concentrations of each amplicon. All five amplicons per participant were pooled, quantified using the Invitrogen Quant-iT Picogreen dsDNA assay (P7589, Invitrogen, Carlsbad, CA, USA) and diluted to 1 ng/μL. Libraries were prepared using the Nextera XT DNA Library Preparation Kit (FC-131-1024, Illumina) and Nextera XT Index Kits (FC-131-1002, Illumina) for amplicon tagmentation and dual-index barcoding, respectively. Indexed amplicons were purified with AMPure XP magnetic beads and a final library consisting of all samples pooled together was diluted to 1.3 ng/µL before sequencing on an Illumina MiSeq. FastQ files were processed using the in-house bioinformatics pipeline MiCall (version 7.15) [41,42]. MiCall can assemble viral genomes by either mapping to a set of reference sequences (which, for the present study, consisted of 114 sequences representing all major HIV subtypes as well as CRF02_AG and CRF06_cpx), or by de novo assembly using the Iterative Viral Assembler (IVA) [43] and Haploflow [44] programs. For samples where de novo assembly produced multiple subgenomic contigs, the pipeline assembled these into a full-genome consensus. Here, plurality consensus sequences from the de novo Haploflow pipeline were used as the primary method for HIV genomic reconstruction, with output from the other assembly methods used to resolve challenging regions. For the resistance analyses, MiCall output summarizing amino acid prevalence at all Protease, RT and Integrase codons was used. Residues present at an intra-host prevalence of ≥5% were considered in resistance analyses.

### 2.4. HIV Subtyping, Phylogenetics, Drug Resistance and Coreceptor Usage Interpretation 

HIV subtype determination was performed using the Recombinant Identification Program (RIP3.0) [45,46,47]. All analyses used a window size of 400 and a 95% confidence threshold (CT). To represent the diversity of HIV strains circulating in Ghana, study sequences were queried against a background alignment of 17 sequences comprising the consensus sequences for subtypes A1, A2, A6, B, C, D, F1, F2, G, H, J, CRF01_AE and CRF02_AG, and reference strains A3.SN.01.DDI579, K.CD.97.97ZR_EQTB11, 06_CPX.AU.96.BFP90 and 09_CPX.GH.96.96GH2911 (as no consensus sequences are available for these) [29]. For protease-RT sequences, the consensus (mixture-containing) Sanger sequences were used for subtyping, while for full-genome subtyping the plurality (non-mixture containing) MiSeq consensus sequence was used. 

Sequences were aligned using MAFFT implemented in HIV Align [48,49], viewed and manually edited in AliView (v1.25). For the protease-RT sequence alignment, 43 codons associated with drug resistance [50] were removed prior to phylogenetic inference so that these residues would not influence tree topology. A maximum likelihood phylogeny was constructed from this alignment using IQTREE [51,52] with automated model selection using ModelFinder [53] and Ultrafast bootstrap option [54]. The tree was visualized and annotated in R (v4.1.2). 

Drug resistance mutation interpretation was performed using the Stanford University HIV Database Program Algorithm version 9.1 (HIVdb) [55,56]. Briefly, the algorithm assigns a score to each mutation associated with decreased susceptibility to a given antiretroviral drug, as well as to specific combinations of mutations. Summed scores determine the sequence’s degree of reduced susceptibility to each drug, where scores between 0–9 denote full susceptibility, 10–14 denote potential low-level resistance, 15–29 denote low-level resistance, 30–59 denote intermediate resistance, and ≥60 denote high-level resistance to a given drug [56]. Here, we considered a sequence as susceptible if its score was between 0–14, and as harboring resistance to a particular drug class (PI, NRTI, NNRTI or INSTI) if its score was ≥15 for at least one drug in the class. For all sequences meeting this threshold, we reported all resistance-associated mutations within it, categorizing these as “major” (mutations that, alone, confer a score of ≥15 to any drug) or “minor/accessory” (mutations that, alone, do not confer clinically relevant resistance).

HIV coreceptor usage was inferred from the V3 loop region within gp120 envelope (env) sequences obtained by MiSeq, using the geno2pheno (g2p) algorithm [57,58] implemented in MiCall. G2p assigns each V3 sequence a “false positive rate” (FPR) value, which represents the likelihood that a CCR5-using virus is misclassified as CXCR4-using. Sequences with low FPR are more likely to be CXCR4-using while those with high FPR are CCR5-using. For each participant, individual complete within-host V3 sequences were reconstructed in MiCall, to generate a list of unique V3 sequences observed per participant. Each of these unique V3 sequences was then interpreted using g2p: as recommended for next-generation sequencing data, unique V3 sequences with FPR <3.5% were denoted as CXCR4-using, while those with FPR ≥3.5% were denoted as CCR5-using. To generate a final coreceptor assignment for each participant, we counted the number of times each unique V3 sequence was observed in the sample (as a proxy for the abundance of this sequence in vivo): a sample was denoted as having CXCR4-using variants if ≥2% of its overall sequences were classified as CXCR4-using; otherwise, it was classified as CCR5-using [59]. 

### 2.5. Statistical Analysis

Associations between categorical variables were determined using Fisher’s exact test or chi-squared test where appropriate using Prism v8.4.3 software (GraphPad). For all comparisons, a two-tailed *p*-value <0.05 was considered statistically significant.

### 2.6. Accession Numbers

GenBank accession numbers for Sanger protease-RT sequences are OP894533–OP894623 while those for Integrase are OP894444–OP894532. Accession numbers for Illumina full-genome HIV consensus sequences are OQ121842–OQ121917.

## 3. Results

### 3.1. Cohort Characteristics

A total of 103 participants were recruited between 2020–2022 from clinics in major cities in Ghana, namely Accra, Elmina and Komenda (Table 1). Of these, 93 (90%) were ART naïve, while 10 (10%) had discontinued first-line ART with no documented treatment failure at least two years prior. A total of 49 (51%) were female, and the overall cohort median age was 38 (interquartile range; IQR 30–49) years. The main mode of infection was heterosexual contact (79%). HIV plasma viral loads (pVL), available for 27 participants, were a median 5.3 [IQR, 4.5–5.9] log_10_ HIV RNA copies/mL. CD4+ T-cell counts were not available.

### 3.2. Subtype Characterization Based on Protease-RT Sequences

**Figure 1:**
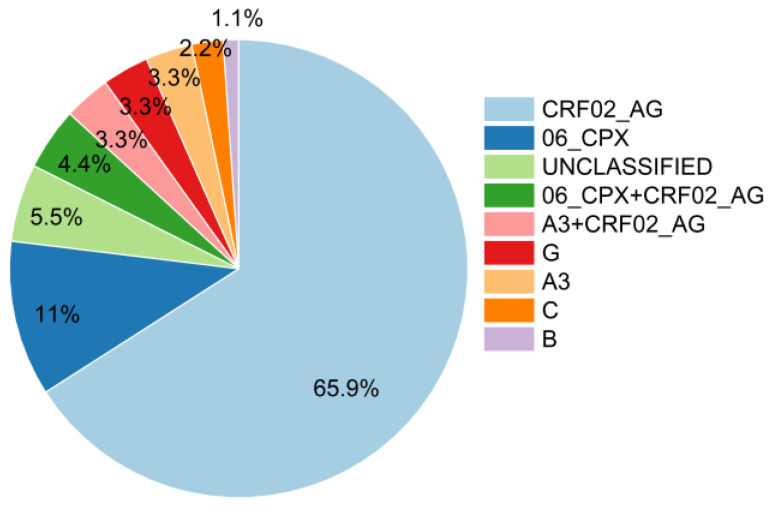
Subtype distribution based on protease-RT sequences.

HIV protease-RT genotyping was successful for 91 participants (88%). As this is the most commonly used region for HIV subtyping, we began by inferring subtype from these data (see methods). Using a RIP window size of 400 and a confidence threshold of 95%, 60/91 (65.9%) of protease-RT sequences were identified as CRF02_AG, with the next most frequent being 06_cpx (10/91; 11%; Figure 1). Next most prevalent were unique recombinant forms that have not yet been described in the literature, including mosaics of 06_cpx and CRF02_AG (4.4%), and recombinants of A3 and CRF02_AG (3.3%). Pure subtypes G (3.3%), A3 (3.3%), C (2.2%) and B (1.1%) were also observed. 

**Figure 2:**
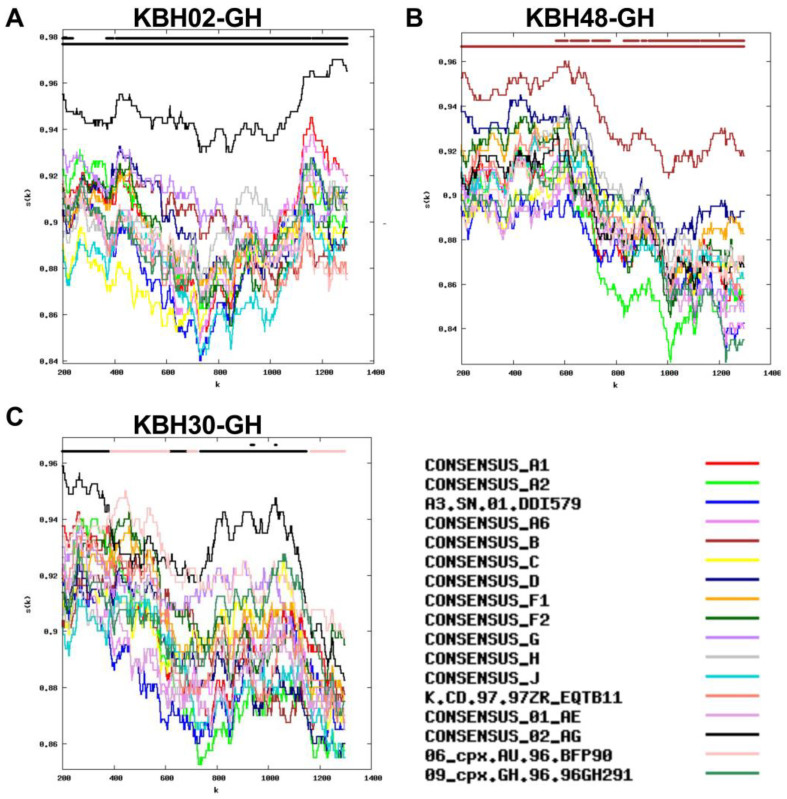
Subtype assignments based on protease-RT sequences. Panels (**A**–**C**): The y-axis denotes the % similarity between the participant sequence to each of 17 reference sequences (each in a different color) over a sliding window of 400 bases (shown on X axis). The bars at the top of each plot indicate the best matching reference sequence over a given sequence region (lower bar) and whether this match meets the 95% confidence threshold (upper bar). Panel (**A**) A “pure” CRF02_AG sequence in participant KBH02-GH. Panel (**B**) Pure subtype B in KBH48-GH. Panel (**C**) A sample that was classified as CRF02_AG based on two short CRF02_AG regions that met the 95% confidence threshold, but that is likely a recombinant of CRF02_AG and 06_cpx (participant KBH30-GH).

Inspection of the protease-RT RIP outputs however indicated that, while some subtype calls were unambiguous (see examples of CRF02_AG and a pure subtype B sequence in Figure 2A,B), others were more uncertain. Participant KBH30-GH’s sequence for example contained only two short CRF02_AG regions that met our predefined 95% confidence threshold, though its RIP plot indicated that it was a likely recombinant of CRF02_AG and 06_cpx (Figure 2C).

**Figure 3:**
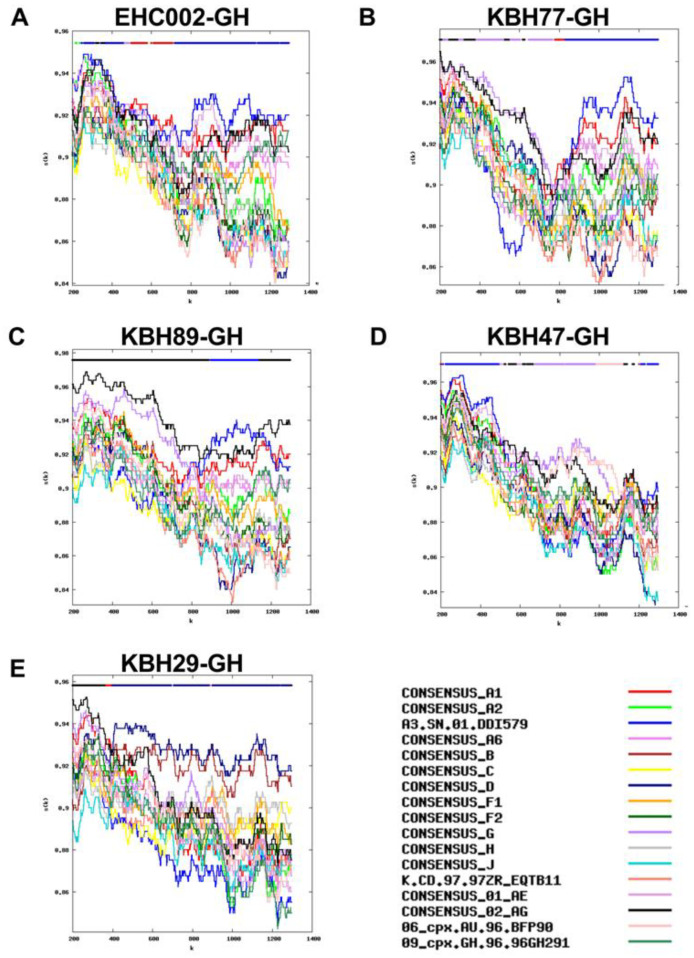
Protease-RT sequences where subtype classification was not possible at the predefined confidence threshold. The y-axis denotes the % similarity between the participant sequence to each of 17 reference sequences (each in a different color) over a sliding window of 400 bases (shown on X axis). The bars at the top of each plot indicate the best matching reference sequence over a given sequence region (lower bar) and whether this match meets the 95% confidence threshold (upper bar). The RIP plots however show the recombinant composition as follows: Panel (**A**) Mosaic of subtypes A3 and A1. (**B**) Mosaic of G and/or CRF02_AG at the 5’ end, with A3 at the 3’ end. Panel (**C**) Likely recombinant of CRF02_AG and A3. Panel (**D**) Mosaic including A-like, G-like, CRF02_AG-like and/or 06_cpx-like sequences. Panel (**E**) Likely recombinant of CRF02_AG and subtype D. The sequences are presented in the same order as they appear in the phylogeny (in Figure 4), from top to bottom.

Related to this, HIV subtype calls could not be determined for 5 (5.5%) of protease-RT sequences, as no part of the sequence matched any reference sequence at the predefined 95% confidence threshold (Figure 3). These are likely unique recombinants, including a mosaic of subtypes A3 and A1 (participant EHC002-GH; Figure 3A), a mosaic of G and/or CRF02_AG at the 5’ end, with A3 at the 3’ end (KBH77-GH; Figure 3B), a likely recombinant of CRF02_AG and A3 (KBH89-GH; Figure 3C), a mosaic including A-like, G-like, CRF02_AG-like and/or 06_cpx-like sequences (though the overlap in the similarity plots makes classification impossible; KBH47-GH Figure 3D) and a likely recombinant of CRF02_AG and subtype D (KBH29-GH; Figure 3E). 

**Figure 4:**
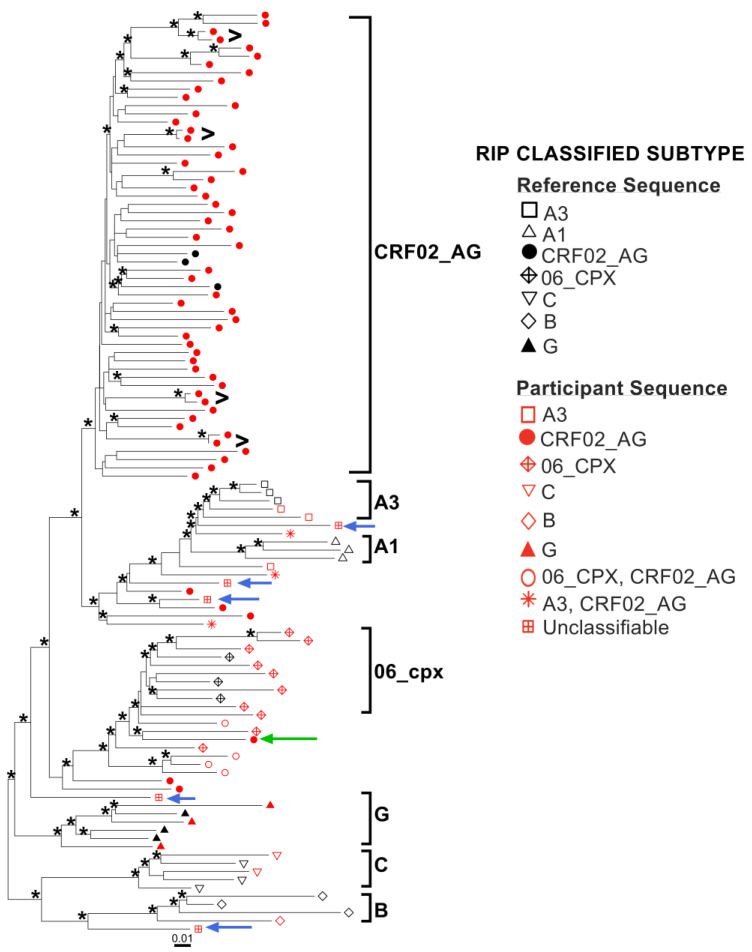
Maximum likelihood protease-RT phylogeny. The tree was inferred from 91 protease-RT sequences from participants (red symbols) and 21 reference sequences representative of cohort diversity (3 each for 7 subtypes; black symbols). Phylogeny is rooted at midpoint. Blue arrows denote sequences with unclassifiable subtypes by RIP, shown in the same order from top to bottom as Figure 3. Black “>“ symbols show known epidemiologically linked pairs. Green arrow shows the sequence in Figure 2C. Scale in estimated nucleotide substitutions per site. Asterisks (*) indicate branches with approximate bootstrap values >70.

We further investigated protease-RT subtypes phylogenetically (Figure 4). Here, the five “unclassifiable” sequences by RIP are shown by blue arrows, and the order in which they appear in the tree from top to bottom matches the order in which they are presented in Figure 3. EHC002-GH (Figure 3A) fell within the broad subclade featuring A1 and A3 sequences, in an intermediate position between A1 and A3 subclades, consistent with it being a recombinant of these two subtypes. Both KBH77-GH (Figure 3B) and KBH89-GH (Figure 3C) were in an intermediate position between the subtype A and CRF02_AG subclades, consistent with them being A/CRF02_AG recombinants. The most complex of the five unclassifiable sequences, KBH47-GH (Figure 3D) clustered close to the internal node giving rise to the CRF02_AG, A and 06_cpx subclades, suggesting that it is a recombinant of these three subtypes. KBH29-GH (Figure 3E) branched off from the internal node giving rise to the subtype B clade, which is consistent with RIP having identified subtype D as the major component of this sequence (subtype D is the most closely related subtype to B). 

The phylogeny also confirmed that KBH30-GH (shown in Figure 2C and indicated in the tree by a green arrow) is likely a novel recombinant comprising CRF02_AG and 06_cpx regions, as it branched near the internal node giving rise to the 06_cpx cluster. In general, however, the tree corroborated the RIP subtype calls in most cases (e.g., see large clade of CRF02_AG sequences matching the CRF02_AG RIP calls; see also the A3/CRF02_AG recombinants and 06_cpx/CRF02_AG recombinants falling within subclades that are intermediate to those containing their parental subtypes). The tree also confirmed four known epidemiologically linked pairs in the cohort (black “>“ symbols).

### 3.3. Subtyping Based on Full HIV Genomes

As the protease-RT fragment represents only ~15% of the total viral genome, it may not fully represent cohort subtype composition. We therefore carried out subtype analysis based on full HIV genome sequences (n = 76, 74%) as this is the gold standard for subtyping in regions with extensive HIV diversity. 

**Figure 5:**
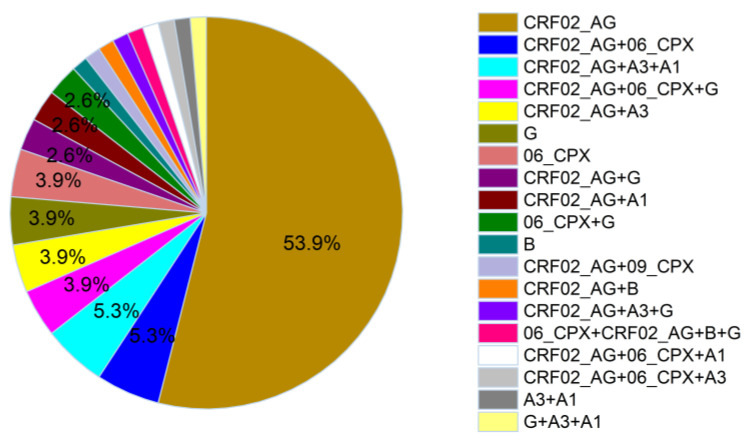
Subtype distribution determined from full HIV genomes (N = 76). Categories indicate subtype composition, not shared breakpoints. Single occurrences are not indicated by percentages.

Based on full-genome HIV sequences, the dominant subtype was CRF02_AG, at 54% prevalence (Figure 5). The next most frequent variants were CRF02_AG-containing recombinants, including CRF02_AG/06_cpx (5.3%), CRF02_AG/A3/A1 (5.3%), CRF02_AG/06_cpx/G (3.9%) and CRF02_AG/A3 (3.9%). In fact, CRF02_AG-containing recombinants represented 31.5% of all HIV genomes sequenced, where the most complex genome comprised regions of 06_cpx, CRF02_AG, subtype B and subtype G (participant KBH34-GH). Only four sequences representing “pure” subtypes (5%) were identified: three subtype G (3.9%) and one B sequence. This subtype B sequence did not have high similarity to any known subtype B reference strains, nor did it closely match any sequence previously deposited in HIV LANL nor any Protease-RT or Integrase sequence recently isolated at the BC Centre for Excellence in HIV/AIDS where the genotyping was performed (unpublished work), supporting its authenticity. The identification of a pure subtype B sequence is notable, as few have been identified in West Africa [8]. Only 1% (13 of 2042) HIV sequences from Ghana in HIV LANL are subtype B, and as of October 2022, the neighboring countries including Togo, Benin and Burkina Faso had reported <1% subtype B prevalence. 

Overall, and as anticipated, full genome subtyping revealed a richer array of recombinant sequences than that estimated using only protease-RT (compare subtypes in Figure 5 to those in Figure 1). When restricted to the 76 participants for whom full genome HIV sequencing was successful, the overall concordance of protease-RT and full-genome-determined subtypes was only 63% (48/76), where discordant calls were the result of either complex recombination patterns in regions outside of protease-RT, or the successful assignment of subtype calls by full genome subtyping in cases where protease-RT-based subtyping yielded no significant result (i.e., unclassifiable sequences shown in Figure 3). In particular, protease-RT subtyping overestimated the prevalence of CRF02_AG by 10% and 06_cpx by 8%. 

**Figure 6:**
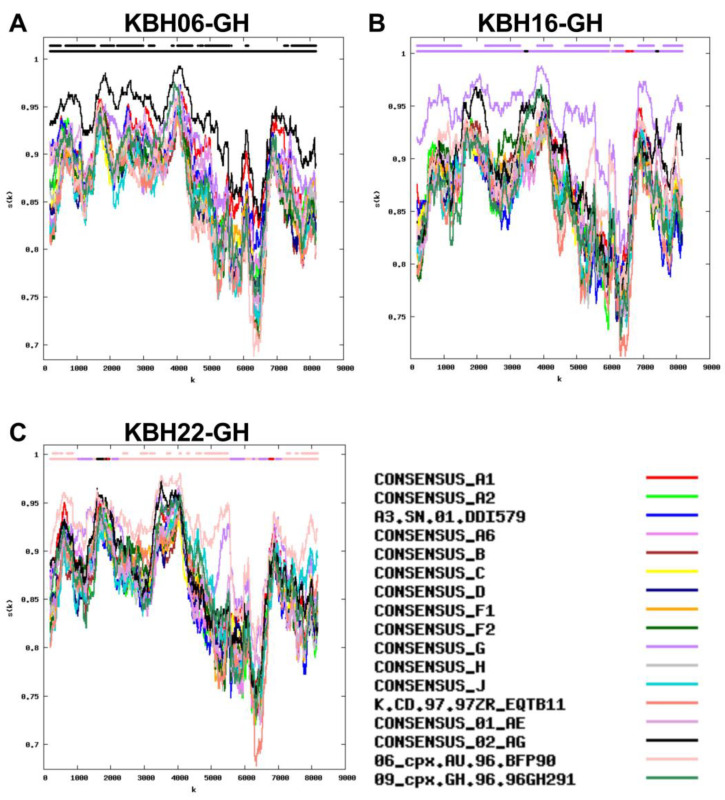
Representative full-HIV-genome similarity plots of major subtypes in our cohort. The y-axis denotes the % similarity between the participant sequence to each of 17 reference sequences (each in a different color) over a sliding window of 400 bases (shown on X axis). The bars at the top of each plot indicate the best matching reference sequence over a given sequence region (lower bar) and whether this match meets the 95% confidence threshold (upper bar). Panel (**A**) CRF02_AG in KBH06-GH. Panel (**B**) Pure Subtype G in KBH16-GH. Panel (**C**) 06_cpx in KBH22-GH.

Representative full-genome similarity plots depicting sequences from three of the most commonly observed subtypes in the cohort are shown in Figure 6.

**Figure 7:**
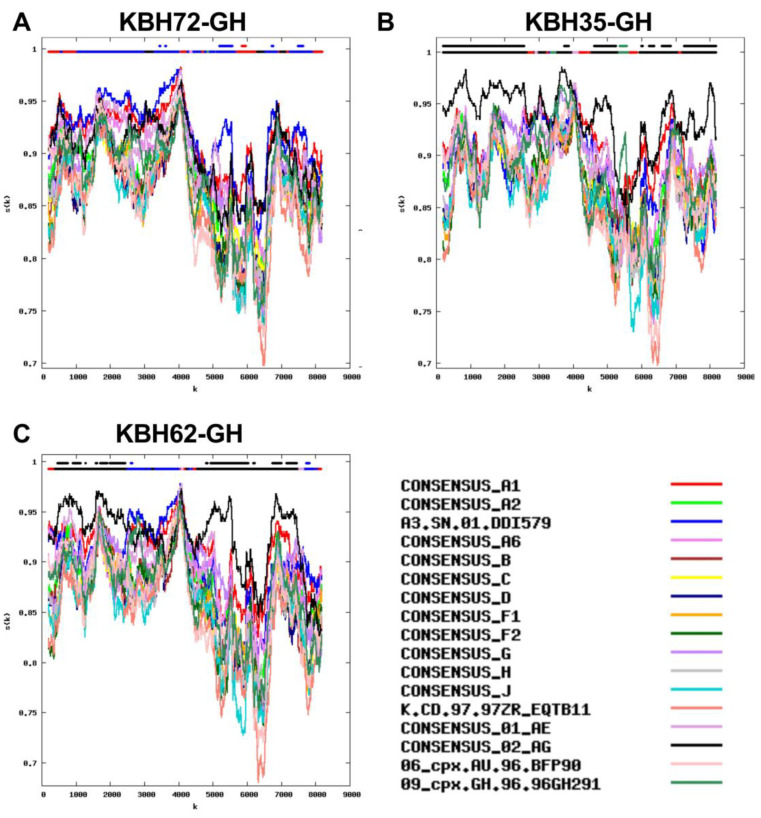
Full-genome similarity plots of unique recombinants. Panels (**A**–**C**): The y-axis denotes the % similarity between the participant sequence to each of 17 reference sequences (each in a different color) over a sliding window of 400 bases (shown on X axis). The bars at the top of each plot indicate the best matching reference sequence over a given sequence region (lower bar) and whether this match meets the 95% confidence threshold (upper bar). Panel (**A**) Novel A3 and A1 recombinant in KBH72-GH. Panel (**B**) Novel recombinant containing CRF02_AG and 09_cpx in KBH35-GH. Panel (**C**) Novel recombinant of CRF02_AG and A3 in KBH62-GH.

Many HIV full genome sequences in our cohort however returned mosaic patterns that were more challenging to interpret, including mosaic patterns that have to date not been described in the Los Alamos HIV database. These included a novel A3/A1 recombinant (Figure 7A), a recombinant containing CRF02_AG and 09_cpx regions (Figure 7B), and recombinants of CRF02_AG and A3 (Figure 7C). 

**Figure 8:**
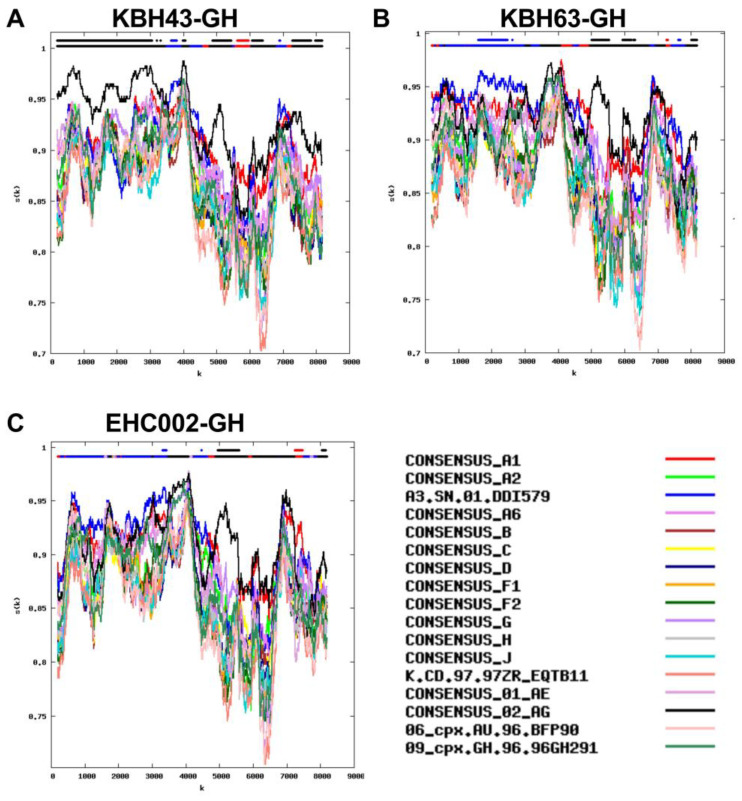
Similarity plots of 3 sequences classified as CRF02_AG/A3/A1 recombinants that do not share common breakpoints, indicating that they arose independently. Panels (**A**–**C**): The y-axis denotes the % similarity between the participant sequence to each of 17 reference sequences (each in a different color) over a sliding window of 400 bases (shown on X axis). The bars at the top of each plot indicate the best matching reference sequence over a given sequence region (lower bar) and whether this match meets the 95% confidence threshold (upper bar). Panel (**A**) CRF02_AG/A3/A1 in KBH43-GH Panel (**B**) CRF02_AG/A3/A1 in KBH63-GH Panel (**C**) CRF02_AG/A3/A1 in EHC002-GH.

These observations confirm that, in global regions where HIV diversity is high, only full-genome HIV subtyping can capture the full picture. Even then, some HIV genomes can remain difficult to classify. Indeed, even the subtype categories listed in Figure 5 do not fully capture the full extent of HIV diversity in the cohort because most of the sequences within a given novel recombinant category do not share common breakpoints, indicating that, while they feature same subtype “components”, they arose independently. An example is shown in Figure 8, where 3 of the 4 samples categorized as CRF02_AG/A3/A1 had distinct recombination breakpoints. Indeed, despite identifying many new unique recombinant forms (URFs) such as these, no URF was observed in more than one participant, further underscoring the extensive regional HIV diversity. 

### 3.4. Drug Resistance 

We investigated drug resistance using Sanger sequencing as the primary genotyping method. We also performed Illumina sequencing to assess concordance with Sanger sequencing, and to investigate the presence of low-abundance resistance mutations [60]. As our cohort comprised both ART-naive individuals and those who had discontinued first-line ART at least two years prior (i.e., individuals who met the WHO definition of “pretreatment resistance”), all participants were grouped together in the drug resistance analysis. Protease-RT genotyping was successful for 91 (88%) participants while integrase genotyping was successful for 89 (86%). 

**Figure 9:**
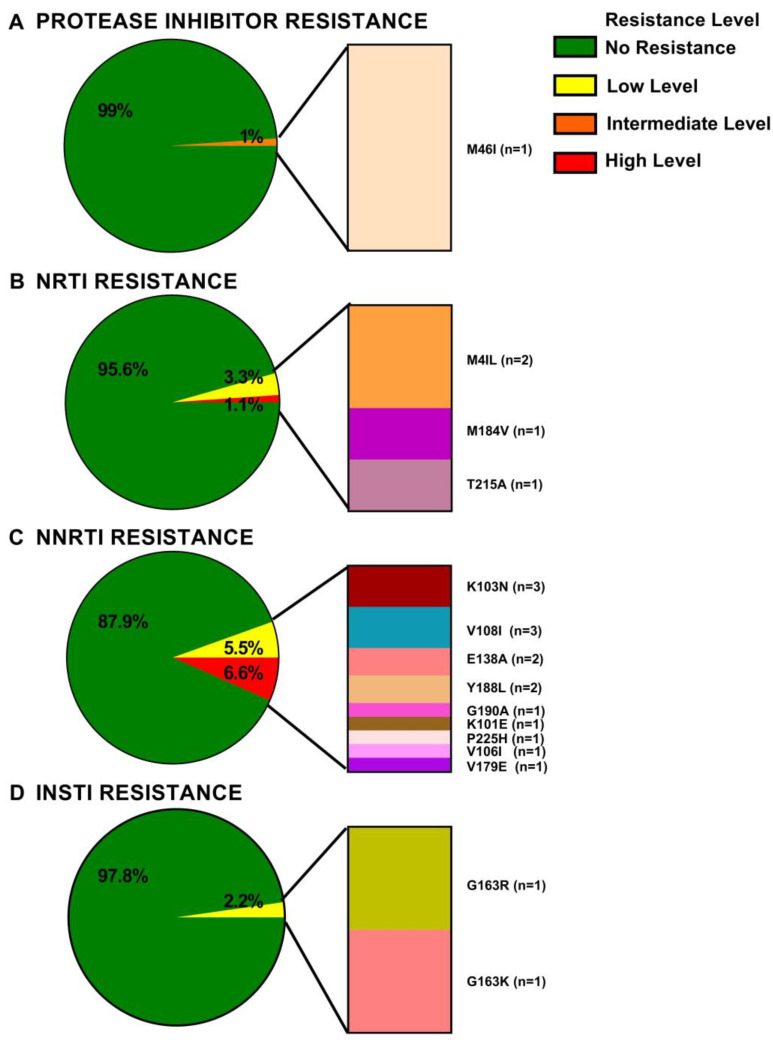
Drug resistance by antiretroviral class, identified by Sanger sequencing. Resistance categories were defined based on the following Stanford scores: Susceptible 0–14, Low-level resistance 15–29, Intermediate resistance 30–59 and High-level resistance ≥ 60. For sequences harboring low, intermediate, or high-level resistance, the individual mutations contributing to the inferred resistance are shown at the right of the pie. Panel (**A**): PI resistance. Panel (**B**) NRTI resistance. Panel (**C**) NNRTI resistance. Panel (**D**) INSTI.

Of these, Sanger sequencing identified 16 participants (17%), 15 ART-naive and one previously treated, whose HIV sequences harboured mutations conferring resistance with a Stanford HIVdb v9.1 score ≥15 to one or more antiretroviral drugs (Figure 9). Of these, 7 (i.e., 7.6% of the cohort overall) harbored intermediate- or high-level resistance to one or more drug. Participants with drug resistance included one individual (1%) with intermediate level protease inhibitor resistance (Figure 9A), 4 (4.4%) with NRTI resistance including some cases of high-level resistance (Figure 9B), 11 (12.1%) with NNRTI resistance including some cases of high level resistance (Figure 9C) and 2 (2.2%) with low level INSTI resistance (Figure 9D). 

These drug resistant HIV sequences harbored the following mutations. A single ART-naive individual harbored the major PI resistance-associated mutation M46I (Figure 9A). The four participants with NRTI resistance-associated mutations (3 ART-naive; 1 previously treated) harbored three major mutations: M41L (observed twice), M184V and T215A (Figure 9B). All NRTI mutations occurred in ART-naive individuals except the M184V. The 11 participants with NNRTI-resistance-associated mutations (10 ART-naive; 1 previously treated) harbored 9 unique mutations. These included the major mutations K103N (observed 3 times, including in the previously treated individual), V108I (n = 3), Y188L (n = 2), E138A (n = 2) and single occurrences of K101E, G190A and P225H. The minor/accessory mutations V106I and V179E were also observed in tandem with the K101E and Y188L in one ART-naive individual (Figure 9C). The two participants with INSTI resistance-associated mutations, both ART-naïve, harbored the G163K and G163R mutations, respectively (Figure 9D).

Of note, three participants, all ART-naïve and harboring CRF02_AG, had either a one (n = 2) or two (n = 1) amino acid insertion following protease codon 35. Insertions at this location are relatively uncommon (only 22/6350 HIV sequences in the Los Alamos database have such an insertion) but are not associated with drug resistance. 

**Figure 10:**
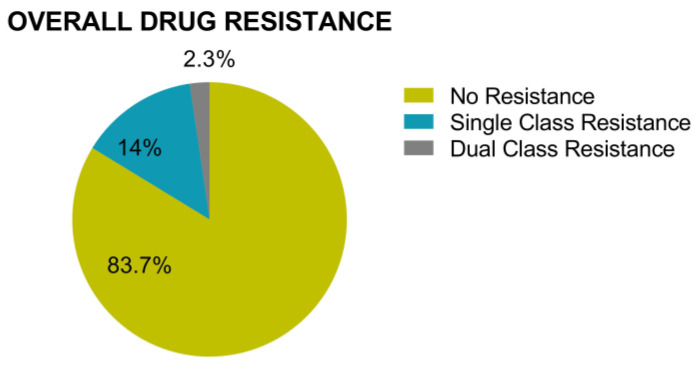
Prevalence of multi-drug resistance, assessed in 86 participants for whom both protease-RT and integrase genotyping was successful. Of the 12 individuals (14%) with single class resistance, 10 had NNRTI resistance, 1 had NRTI resistance, 1 had INSTI resistance. Two cases of dual-class resistance were to NRTI/NNRTI and NRTI/INSTI, respectively.

Both protease-RT and Integrase genotyping was successful for 86 participants, allowing us to also investigate multi-class drug resistance in this subset (Figure 10). Among this group, 12 participants (14%), all of whom were ART- naïve, harbored single class resistance. These included one case of low-level NRTI resistance (to zidovudine [AZT] and stavudine [d4T]), one case of low-level INSTI resistance (to elvitegravir [EVG] and raltegravir [RAL]) and 10 cases of NNRTI resistance. The latter included 5 instances of low-level resistance (3 to nevirapine [NVP] and 2 to rilpivirine [RPV]) and 5 cases of high-level NNRTI resistance. Two participants (2.1%) exhibited dual class resistance. The first, an ART-treated individual, had high-level resistance to the NRTIs abacavir [ABC], emtricitabine [FTC] and lamivudine [3TC] as well as the NNRTIs efavirenz [EFV] and NVP, while the second, an ART-naive individual, had low level resistance to the NRTIs AZT and D4T and the INSTIs EVG and RAL. It is important to note that the observed pretreatment resistance included resistance to the previously preferred NNRTI-based first-line regimens in Ghana, but not to the INSTI dolutegravir (DTG), which was introduced as preferred first-line treatment in 2019 [25]. 

We next investigated resistance prevalence by HIV subtype. After classifying sequences into four subtype categories (CRF02_AG, pure subtypes, 06_cpx and “other”, where the latter includes unique recombinants and samples with no significant subtype in protease-RT or integrase), we observed no association between HIV subtype and drug resistance in either protease-RT (*p* = 0.79) or integrase (*p* = 0.36). 

As Sanger sequencing cannot reliably identify low-abundance HIV variants that are present below ~20–25% within-host frequency [61], we compared mutation patterns identified via Sanger and Illumina (MiSeq) sequencing in the subset of 86 participants for whom MiSeq resistance determination was successful (all of whom also had Sanger data). In this subset, mutations conferring single- and dual-class resistance were observed in 14 (16%) and 2 (2.2%) participants. Importantly, all mutations found in MiSeq data at >15% frequency were identified by Sanger, indicating a 100% concordance at this threshold. However, MiSeq identified 7 additional participants who harbored mutations that confer decreased susceptibility to one or more antiretroviral drugs at 5–15% within-host frequency, that were not detected by Sanger sequencing (Supplementary Table S4). These included one participant (EHC003-GH) for whom both Sanger and MiSeq had identified the major NNRTI-resistance mutation E138A in reverse transcriptase, but where MiSeq additionally identified M230I, which confers intermediate resistance to NVP and RPV, at 7.6% within-host prevalence. It also included six additional participants for whom Sanger sequencing had not identified any resistance mutations, but for whom MiSeq identified a low-abundance variant. These included one participant (KBH10-GH) with a MiSeq-identified F53L mutation in protease, which confers low-level resistance to saquinavir (SAQ), at 6.4% within-host prevalence. It also included two participants (KBH43-GH and CHC003-GH) with the integrase mutation G140R that confers intermediate resistance to RAL and EVG and high-level resistance to cabotegravir (CAB), at 6.3% and 8.7% within-host frequencies. In two additional participants (KBH94-GH and KBH90-GH), MiSeq detected the E138A mutation in reverse transcriptase that confers low-level RPV resistance at 5.3% and 13% within-host frequency, respectively. Finally, in participant (KBH70-GH) MiSeq detected the “revertant” T215S mutation associated with low-level resistance to AZT at a 6.4% within-host frequency. 

As such, if resistance genotyping had been performed by MiSeq and all within-host variants >5% had been included in the interpretations, the overall resistance prevalence would have been 25%, compared to 17% as determined by Sanger. Specifically, single-class resistance prevalence estimates would have increased from 14% (Sanger) to 23% (MiSeq), while dual-class resistance prevalence estimates would not have changed. 

### 3.5. Coreceptor Usage

**Figure 11:**
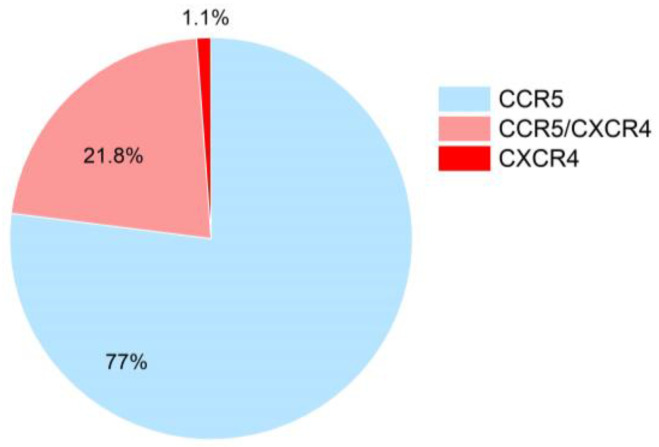
Coreceptor usage based on V3 loop sequences genotyped using Illumina MiSeq. Coreceptor usage was inferred using the g2p algorithm. A sample was denoted as containing CXCR4-using variants when ≥2% of its g2p scored reads had a false positive rate (FPR) of ≤3.5%.

We determined HIV coreceptor usage by analyzing individual unique within-host envelope V3 loop sequences recovered from Illumina sequencing of the gp120 region, using the geno2pheno (g2p) algorithm (Figure 11). Of the 87 participants for whom gp120 sequencing was successful, 67 (77%) harbored exclusively CCR5-using variants. A further 19 (21.8%) harbored a mixture of viruses capable of cell entry via the CCR5, CXCR4 and/or both coreceptors. In these participants, CXCR4-using viruses represented a median of 24% (IQR 11–71%) of their within-host viral populations. One individual, an ART naïve participant, harboured a pure CXCR4-using viral population.

Finally, we investigated associations between coreceptor usage and env subtype (CRF02_AG, pure subtype, 06_cpx and “other” determined using RIP from the gp120 MiSeq consensus sequence) in 85 persons for which we successfully sequenced the entire gp120 region. Overall, we observed no statistically significant association between coreceptor usage and subtype (Chi-squared *p* = 0.47). The one case of pure CXCR4 usage was observed in a participant with CRF02_AG. 

## 4. Discussion

We characterized HIV subtype diversity (using both protease-RT and full-genome HIV sequences), drug resistance and predicted coreceptor usage in a cohort of predominantly (90%) ART-naïve persons in Ghana. Though our cohort was relatively modest in size, participant characteristics were nevertheless consistent with the epidemiology of HIV in Ghana. Our cohort comprised slightly more females than males, consistent with the over-representation of females among PLWH globally (UNAIDS estimates that 54% of all PLWH in 2021 were women and girls [2]), and in sub-Saharan Africa [62,63,64], including Ghana [16,27,65], in particular. Consistent with previous reports from Ghana [19,27,66], the dominant mode of transmission in our cohort was heterosexual, and the cohort age distribution was comparable to recent studies in the region [25].

Our results confirm that protease-RT-based HIV subtyping, though routinely performed, does not fully capture HIV subtype diversity in regions with high population-level HIV diversity, such as Ghana [30]. Though both protease-RT and full-genome HIV subtyping identified CRF02_AG as the dominant variant in Ghana, protease-RT-based subtyping overestimated CRF02_AG prevalence by over 10% relative to whole-genome sequencing (66% vs. 54%, respectively). Indeed, overall concordance between protease-RT and full-genome-based HIV subtyping was only 63%, where discordant calls were attributable to additional recombinant complexity that either occurred outside of protease-RT, or that could not be resolved within this sub-genomic region at our predefined confidence threshold.

Full-genome HIV subtyping also revealed a large proportion of novel recombinants that have not previously been described, including mosaics of CRF02_AG and/or cpx_06 along with other subtypes, that together made up nearly 37% of full-genome sequences in our cohort. Of note, most of these recombinants had unique breakpoints, indicating that they had arisen independently and were not the result of shared transmission within the cohort. 

Importantly, our estimate of 54% CRF02_AG prevalence based on full-genome sequencing is substantially lower than that currently reported for Ghana (as of mid-November 2022, the Los Alamos HIV database estimates CRF02_AG prevalence at 78%; with 1254 of 1609 Ghanaian sequences being CRF02_AG [8]). This discrepancy is not due to our use of full-genome (rather than subgenomic) subtyping, as even our protease-RT-based subtyping estimated CRF02_AG prevalence at 66%. Instead, our results indicate that HIV genetic diversity in Ghana may be substantially higher than current estimates: specifically, that “pure” CRF02_AG prevalence is considerably lower than currently reported, while the prevalence of novel recombinants is considerably higher.

Of note, CRF02_AG is estimated to be the most prevalent HIV recombinant strain globally (7.7%) [4], despite its relative restriction to West Africa [67]. Though the reasons for CRF02_AG’s spread are unclear (and could largely be due to founder effects), a 2004 study from Ghana reported that asymptomatic individuals with CRF02_AG had fivefold higher viral loads than those with other subtypes, suggesting a replicative advantage [68], a hypothesis that is supported by a recent report suggesting that CRF02_AG has a higher in vitro replicative capacity relative to its parental subtypes [69]. Regardless, our frequent observance of CRF02_AG along with unique recombinants, many of which contain CRF02_AG, is consistent with the ongoing generation and spread of HIV recombinant forms which now make up 23% of HIV infections globally [4]. Indeed, the high prevalence of URFs observed in this study is consistent with previous reports from Ghana [30,34,70]. High URF prevalence in the region is likely attributable to multiple factors, including high HIV subtype diversity in West Africa as well as socio-epidemiological factors. Due to the stigma associated with HIV, many individuals remain unaware of their status, and barriers to treatment access remain [71,72]. There are also high levels of migration, including among populations at increased risk of HIV [73]. Together, these factors contribute to high rates of multiple or superinfection [70], which increases the likelihood that novel recombinants will form. 

Our results also enhance our understanding of pretreatment drug resistance in Ghana. Using Sanger sequencing, which can reliably detect minority HIV variants at a threshold of about 20–25% of the within-host viral population, and is still widely used for HIV drug resistance genotyping globally [74,75], we observed a pretreatment drug resistance prevalence of 17% (16/94). This total included 9 individuals (9.6%) with resistance to one or more drugs used in recommended first- or second-line regimens. NNRTI resistance was by far the most commonly observed type of resistance, at 12% prevalence. Specifically, we observed three instances of the major resistance mutations K103N (commonly selected in persons receiving EFV or NVP [76,77] and whose presence increases the probability of virological failure of common NNRTI-based WHO first-line regimens [78,79]) and V108I. We also observed two instances each of Y188L and E138A, and single occurrences of K101E (observed in tandem with Y188L in an ART-naive person), G190A and P225H (observed in tandem with K103N in an ART-naive individual). NRTI, PI and INSTI resistance was less common, observed at 4.4%, 1% and 2.2% prevalence, respectively. The relatively low prevalence of INSTI resistance supports the recent shift towards use of INSTI-based regimens as first-line therapy in Ghana [25]. Most cases of pretreatment resistance were limited to single-class resistance. Dual-class pretreatment resistance was uncommon (2.3%), and no participant exhibited triple or quadruple-class resistance. Of note, Illumina sequencing identified an additional seven individuals harboring minority (5–15% within-host prevalence) variants that were not detected by Sanger sequencing, including 2 cases where a minority variant was associated with high-level resistance (e.g., G140R in KBH43-GH which leads to high level CAB resistance).

Nevertheless, the high concordance between the two sequencing methods demonstrates the continued relevance of Sanger sequencing for drug resistance genotyping. Though the detection of low-abundance resistance mutations in this population is notable, the relevance of these mutations to treatment outcomes remains unclear. While some prior studies have demonstrated associations between low-abundance (<15% within-host prevalence) mutations—in particular minority NNRTI resistant variants [80]—and poorer virologic outcomes in ART-naïve individuals, other studies have failed to demonstrate any impact on clinical outcomes [81,82]. The impact of minority variants on PI- or INSTI-based regimens has not been established. Further studies are required to elucidate the impact of low-abundance variants on antiretroviral treatment outcomes, and the potential added benefit of incorporating deep-sequencing approaches for HIV drug resistance into routine clinical management or population-level surveillance [83].

While CCR5-using viruses are preferentially transmitted and typically predominate during early infection [38], available data suggest that 6–18% of individuals in early infection may harbor CXCR4-using variants [84,85]. Broadly consistent with this, 23% of study participants harbored CXCR4-using variants, though most would have likely already reached the chronic phase of infection at study enrolment, despite their ART-naive status. Coreceptor usage may also differ between subtypes and CRFs [38,86]. Intriguingly, a study undertaken in neighboring Guinea Bissau reported 86% CXCR4 tropism in 111 CRF02_AG sequences from participants in late stage infection [87], suggesting that CXCR4 usage may occur more frequently in CRF02_AG, particularly as the infection progresses. In the present study, however, we did not observe any association between HIV subtype and coreceptor usage. That said, when comparing coreceptor usage findings across the literature, it is important to keep in mind that direct comparisons cannot always be made, since different studies use different methods, interpretation algorithms and cutoffs. 

Our study has some limitations. Sociodemographic data were collected by self-report, as were data on treatment history. Date of HIV infection, prior ART regimen (for the ART experienced subset) and CD4+ T-cell counts data were not available, while plasma viral loads were available for less than one-third of the cohort. HIV sequences were bulk-amplified without the use of unique molecular identifiers, so our estimates of within-host drug resistance mutation prevalence, as well as our estimates of within-host X4 co-receptor usage prevalence, should be interpreted with caution as they may not reflect true within-host variant prevalence. We note however that the g2p cutoffs that we used to identify within-host X4 sequences were those that were defined in the original study that validated deep V3 sequencing as an accurate method to genotypically infer HIV-1 co-receptor usage, a study that also did not employ unique molecular identifiers during HIV genotyping [59]. As coreceptor usage was inferred from unique V3 loop sequences excised from env-gp120 sequences rather than direct amplification of the much smaller V3 loop region, it is possible within-host V3 diversity was underestimated as full gp120 amplification may have been less efficient. The g2p algorithm has also been reported to be less sensitive in some non-B subtypes including CRF02_AG [88,89], which could impact coreceptor usage predictions.

## 5. Conclusions

Our study of HIV-1 subtype diversity (from full viral genomes), drug resistance and coreceptor usage is the first of its kind to be undertaken for Ghana. We demonstrated that CRF02_AG is the dominant subtype in circulation (54%), with unique recombinant forms containing CRF02_AG, cpx_06 and/or other subtypes also present at considerable (nearly 37%) prevalence. This frequent observation of unique recombinant forms strongly suggests that HIV-1 superinfection is not uncommon [90] and this is leading to the ongoing generation of novel complex recombinant viruses in the region. This highlights the importance of public education on HIV prevention measures, the importance of regular HIV testing, and the expansion of antiretroviral treatment to reduce disease progression and transmission risk. Our characterization of 17% pretreatment drug resistance prevalence (including 12% pretreatment resistance to NNRTIs) in this mainly ART-naïve cohort contributes important data to guide population-level HIV treatment recommendations and supports the recent decision to transition to dolutegravir-based first line regimens. Ultimately, our findings underscore the importance of continued HIV molecular surveillance in resource-limited regions to inform treatment strategies to improve the health of people living with HIV. 

## Figures and Tables

**Table 1:** Participant Characteristics.

Sex at birth (n = 96 *)	
Male, n (%)	47 (49%)
Female, n (%)	49 (51%)
Age in years (n = 96 *)	38 (30–49)
Males, median (IQR)	41 (31.5–48.5)
Females, median (IQR)	36 (29–52)
ART Status (n = 103)	
ART Naïve, n (%)	93 (90%)
ART previously discontinued, n (%)	10 (10%)
Infection Risk Group (n = 103)	
Heterosexual, n (%)	81 (79%)
Men who have sex with Men, n (%)	1 (1%)
Vertical Transmission, n (%)	1 (1%)
Sharps, Needle, n (%)	4 (4%)
Unknown/Unsure, n (%)	16 (15%)
Plasma viral load (n = 27 *)	
median (IQR) Log_10_ HIV RNA copies/ml	5.3 (4.5–5.9)

* Sociodemographic and clinical data were unavailable for some participants; Ns with available data are indicated.

## Data Availability

Sequence data have been deposited in GenBank. GenBank accession numbers for Sanger protease-RT sequences are OP894533–OP894623 while those for Integrase are OP894444–OP894532. Accession numbers for Illumina full-genome HIV consensus sequences are OQ121842–OQ121917.

## Supplementary Materials

The following supporting information can be downloaded at https://www.mdpi.com/article/10.3390/v15010128/s1. Table S1: Primary PCR Primers; Table S2: Alternate PCR Primers; Table S3: Sanger Sequence Primers for Polymerase Region; Table S4: Clinically Relevant Drug Resistance Mutations Observed by Illumina Sequencing.
