# Supplementary material for: Molecular Epidemiology of HIV-1 in Ghana: Subtype Distribution, Drug Resistance and Coreceptor Usage
Source: Viruses. 2022 Dec 31;15(1):128. doi: 10.3390/v15010128 (PMC9865111; doi:10.3390/v15010128)
Supplement: Supplementary file 1 [file viruses-15-00128-s001.zip › viruses-2105205-supplementary.pdf]

Primary and alternative ("back-up") primers used for PCR, and those used for sequencing, are detailed in the below tables:

Table S1: Primary PCR Primers

REACTION	PRIMER NAME	DIRECTION	HXB2 NUC COORD - 5’	HXB2 NUC COORD - 3’	SEQUENCE
Gag-pr-1stF	623-Fi	F	623	649	AAATCTCTAGCAGTGGCGCCCGAACAG
Gag-pr-1stR	2cRx_CRF02	R	2849	2824	TAATCCYGCGGGATGAGGTATTCCTA
Gag-pr-2ndF	Gag+1_CRF02	F	671	697	TCCAGAGAAGMTCTCTCGACGCARGGAC
Gag-pr-2ndR	ProA_CRF02	R	2735	2709	GGCAAATAYTGGAGTATTRTATGGRTT
Vpr-gp120-1stF	VP5445F_CRF02	F	5445	5471	TCMAGCAGGACATAATAAGGTAGGATC
Vpr-gp120-1stR	CO602_CRF02	R	7817	7788	GCCCATAGTGCTTCCTGCTGCTCCYARGAA
Vpr-gp120-2ndF	VP5550F_CRF02	F	5550	5574	AGAAGAYAGATGGAACRAGCCCC
Vpr-gp120-2ndR	V3CR_CRF02	R	7760	7736	TGCTCTTTTTTCTCTCYSCACYACT
Pr-rt-1stF	5CP1_CRF02	F	1981	2008	GAAGGACACCTAGCCAGAAATTGCARRG
Pr-rt-1stR	RT3.1_CRF02	R	3859	3830	GCTCCTACTATGGGKTCTTTYTCTAAYTG
pr-rt-2ndF	2.5_CRF02	F	2011	2039	CCTAGGAAAARGGGCTGTTGGAAATGTGG
pr-rt-2ndR	RT3799R_CRF02	R	3799	3777	ACAAACTCCCATTCAGGRATCCA
gp41-nef-1stF	GP41Fo_CRF02	F	7626	7648	TTCAGGCCTGGAGGAGGARAYAT
gp41-nef-1stR	3LTRi	R	9628	9603	TCAAGGCAAGCTTTATTGAGGCTTAA
gp41-nef-2ndF	GP41Fi_CRF02	F	7650	7674	AGGGACAATTGGAGAAGTGAATTRT
gp41-nef-2ndR	3UTRi_CRF02	R	9592	9615	TATTGAGGCTTAAGCAGTGGGTTC
rt-vpu-pcr-primers-new1st	RT3536F_CRF02	F	3536	3560	ACAGAAACAAGGGCARGACARTGG
rt-vpu-1stR	SC05R_CRF02	R	6004	5982	AGTTCCTCGTCGGYGTCTCCRCT
rt-vpu-pcr-primers-new2nd	RT3615F_CRF02	F	3615	3640	AAAARGAGGTCTGCYCACACTAATGA
rt-vpu-2ndR2	SC02R_CRF02	R	5980	5957	CTTCCTGCCATAGGAGATGCCTAA
Integrase-1stF	RT4025F_AG	F	4025	4048	AGTAAAYATAGTAACAGACTCACA
Integrase-1stR	IN5285R_AG	R	5285	5309	CCCAAKTGCCAGTCTYTTTCTCCTG
Integrase-2ndF	RT4035F	F	4035	4060	GTAACAGACTCACAGTATGCATTAGG
Integrase-2ndR	IN5267R	R	5267	5292	TCTCCTGTATGCARACCCCAATATGT

HXB2 is the reference HIV-1 genome, derived from one of the earliest clinical isolates used generally for HIV genome annotation. Here, we indicate primer location relative to the whole genome HIV sequence with DNA synthesis occurring in the 5’-3’.

Table S2: Alternative ("Back-Up") PCR Primers

REACTION	PRIMER NAME	DIRECTION	HXB2 NUC COORD - 5’	HXB2 NUC COORD - 3’	SEQUENCE
rt-vpu-pcr-primer	RT3297F_CRF02	F	3297	3322	GAMAGCTGGACTGTCAATGATATACA
rt-vpu-pcr-primers-1STR	SC05R_CRF02	R	6004	5982	AGTTCCTCGTCGGYGTCTCCRCT
rt-vpu-pcr-primers-new2nd F	RT3578F_CRF02	F	3578	3604	AGAGCCATWYAAAAATCTAAAAACAGG
rt-vpu-pcr-primers-2ND R	SC02R_CRF02	R	5980	5957	CTTCCTGCCATAGGAGATGCCTAA
pr-rt-1stF	5CP1_CRF02_2	F	1981	2008	GAAGGACACCTAGCCAGAAATTGCARGG
-pr-rt-1stR	RT3810R_CRF02	R	3810	3789	GAGGGGTATTGACAAAYTCCCA
-pr-rt-2ndF	2.5_CRF02	F	2011	2039	CCTAGGAAAARGGGCTGTTGGAAATGTGG
-pr-rt-2ndR	RT3799R_CRF02	R	3799	3777	ACAAACTCCCATTCAGGRATCCA
-gp41-nef-1stF	GP41Fo_CRF02_WD	F	7626	7648	TTCAGRCCTGGAGGAGGAGAYAT
-gp41-nef-1stR	R9662-9686	R	9686	9662	TGAGGGATCTCTAGTTACCAGAGTC
-gp41-nef-2ndF	GP41FI	F	7652	7674	GGACAATTGGAGAAGTGAATTAT
-gp41-nef-2ndR	R9604-9632	R	9632	9604	GCACTCAAGGCAAGCTTTATTGAGGCTTA
-gag-pr-1stF	623-Fi	F	623	649	AAATCTCTAGCAGTGGCGCCCGAACAG
-gag-pr-1stRDR	ProA_CRF02_2	R	2735	2713	GGCAAATATTGGAGTRTTRTATG
-gag-pr-2ndF	GS1F_CRF02	F	688	709	GACGCARGGACTCGGCTTGCTGA
-gag-pr-2ndRdR	ProC_CRF02	R	2724	2697	GAGTRTTGTATGGATTTTCAGGCCCAAT
-vpr-gp120-1stF	VPR5438_CRF02	F	5438	5466	GTGAATATCAARCAGGACATAAYAAGGTA
-vpr-gp120-1stR	CO602_CRF02_2	R	7817	7788	GCCCATAGTGCTTCCTGCTGCTCCYAAGAA
-vpr-gp120-2ndF	VPR5557_CRF02	F	5557	5580	AGATGGAACRAGCCCCAGAAGACC
Integrase-1stF	4059F	F	4059	4080	GGAATCATTCAAGCACAACCAG
Integrase-1stR	IN5267R	R	5267	5293	TCTCCTGTATGCARACCCCAATATGT
Integrase-2ndF	IN4164F	F	4164	4189	CACAAAGGAATTGGAGGAAATGAACA
Integrase-2ndR	IN5214R	R	5214	5216	TGGGATGTGTACTTCTGAACTTA

Table S3: Sanger Sequence Primers (Polymerase Region)

REACTION	PRIMER NAME	DIRECTION	HXB2 NUC COORD - 5’	HXB2 NUC COORD - 3’	SEQUENCE
Protease/RT	1F_CRF02	F	2142	2162	CAGACCARARCCAACAGCYCC
Protease/RT	2FOR	F	2696	2713	AATTGGGCCTGAAAATCC
Protease/RT	5B_CRF02	R	2839	2820	GGATGAGGTATTCCTARYTG
Protease/RT	RH14_CRF02	F	2930	2956	TACTGCATTYACTATACCTAGTRTAAA
Protease/RT	BR	R	3019	3003	GGTGATCCTTTCCATCC
Protease/RT	RT3208F_CRF02	F	3208	3229	ARCATCAGAARGAACCYCCATT
Protease/RT	NE1_CRF02	R	3303	3333	CCACTAATTTCTGTATRTCATTGACAGTCCA
Protease/RT	1B_CRF02	R	3794	3778	YTCCCAYTCAGGAATCC
Integrase	IN4141F	F	4141	4165	TCTACCTGTCATGGGTACCAGCACA
Integrase	IN4430R_CRF02	R	4430	4407	ATGTGTACAATCTAATTGCCATAT
Integrase	IN4452F	F	4452	4474	GTAGCAGTCCATGTAGCCAGTGG
Integrase	IN4801R	R	4801	4776	ATCCCCCCTTTTCTTTTAAAATTGTG
Integrase	IN5214R_CRF02	R	5214	5192	TGGRATGTGTACTTCTGAACTTR
Integrase	INS1F_CRF02	F	3685	3705	GCATAGTAATATGGGGAAAGA
Integrase	RT3678F_CRF02	F	3680	3699	AGAAAGCATAGTAATATGGG
Integrase	VIF4888F_CRF02	F	4888	4912	TTCAAAATTTTCGGGTTTATTWCAG

Table S4: Clinically Relevant Drug Resistance Mutations Observed by Illumina Sequencing

PROTEASE

PARTICIPANT	*SUBTYPE*	*TREATMENT STATUS*	*MUTATION (within-host prevalence in MiSeq data)*	*CONFERS RESISTANCE TO*
KBH10-GH	CONSENSUS_02_AG	ART NAIVE	F53L* (0.064)	SQV
KBH11-GH	CONSENSUS_02_AG	ART NAIVE	M46I (0.335)	NFV

REVERSE TRANSCRIPTASE

PARTICIPANT	*SUBTYPE*	*TREATMENT STATUS*	*MUTATION (within-host prevalence in MiSeq data)*	*CONFERS RESISTANCE TO*
KBH13-GH	CONSENSUS_02_AG	ART NAIVE	K103N (0.997), P225H (0.995)	EFV, NVP, DOR
KBH15-GH	CONSENSUS_02_AG	ART NAIVE	K101E (0.225), V106I (0.728), V179E (0.639), Y188L (0.996)	DOR, EFV, NVP, RPV, ETR
KBH20-GH	CONSENSUS_02_AG	ART NAIVE	V108I (0.187)	NVP
KBH25-GH	CONSENSUS_02_AG	ART NAIVE	E138A (0.998)	RPV
KBH40-GH	CONSENSUS_C	ART EXPERIENCED	M184V (0.997)<br>K103N (0.996)	ABC, FTC,3TC, EFV, NVP
KBH42-GH	06_CPX	ART NAIVE	M41L (0.997)	AZT, D4T
KBH57-GH	CONSENSUS_02_AG	ART NAIVE	V108I (0.708)	NVP
KBH61-GH	CONSENSUS_02_AG	ART NAIVE	Y188L (0.981)	DOR, EFV, NVP, RPV
KBH62-GH	A3, CONSENSUS_02_AG	ART NAIVE	K103N (0.743)	EFV, NVP
KBH70-GH	CONSENSUS_02_AG	ART NAIVE	T215S* (0.064)	AZT, D4T
KBH77-GH	Unclassifed	ART NAIVE	G190A (0.994)	EFV, NVP, RPV
KBH78-GH	06_CPX	ART NAIVE	M41L (0.995)	AZT, D4T
KBH85-GH	CONSENSUS_02_AG	ART NAIVE	V108I (0.985)	NVP
KBH89-GH	Unclassifed	ART NAIVE	T215A (0.812)	AZT, D4T
KBH90-GH	CONSENSUS_02_AG	ART NAIVE	E138A* (0.13)	RPV
KBH94-GH	CONSENSUS_02_AG	ART NAIVE	E138A* (0.053)	RPV
EHC003-GH	CONSENSUS_02_AG	ART NAIVE	E138A (0.926), M230I* (0.076)	RPV,DOR,EFV,ETR,NVP

INTEGRASE

PARTICIPANT	*SUBTYPE*	*TREATMENT STATUS*	*MUTATION (within-host prevalence in MiSeq data)*	*CONFERS RESISTANCE TO*
KBH31-GH	CONSENSUS_02_AG	ART NAIVE	G163R (0.584)	EVG, RAL
KBH43-GH	A3, CONSENSUS_02_AG	ART NAIVE	G140R* (0.063)	RAL, EVG, CAB
KBH78-GH	06_CPX	ART NAIVE	G163K (0.997)	EVG, RAL
CHC003-GH	CONSENSUS_02_AG	ART NAIVE	G140R* (0.087)	RAL, EVG, CAB

*Mutations prevalent at < 15% in MiSeq data, denoted by asterisks, were not observed in Sanger sequence data. For mutations that confer resistance to a given drug with a Stanford HIVdb score of 15 or higher, the drug is listed in the final column.
